# Metals and Alloys

**Published:** 1863-01

**Authors:** B. Wood

**Affiliations:** New York


### METALS AND ALLOYS.

BY DR. B. WOOD.

(Continued from p. 536, Vol. XVI.)

### *Bismuth.*

Bismuth, formerly styled a "semi-metal," and known among artizans as tin glass, is a brittle metal, lamellar or crystalline in texture, of a tawney purplish "white" color, or more strictly, a bright purplish gray with a faint yellowish tint. It breaks with a crystalline fracture, presenting a glistening appearance. It may be reduced a little without breaking, under the hammer, and is slightly flexible:—it is not so brittle as antimony but is more fragile, that is, it breaks with less force. The facets of its crystals are larger, equally brilliant, but not so white, and exhibit a sort of iridescence. Its hardness is differently stated in the books. In some tables it is placed above zinc, and between gold and silver. If pure, it is quite soft, ranking on the scale, given in the first of these papers, one degree harder than tin, and two degrees less than cadmium. As usually obtained in commerce, owing to contamination with other metals, antimony, copper, arsenic, etc., it is considerably harder, often as much as two degrees. If, however, the admixture consists of lead, the hardness is not increased.

The nature of the admixtures may be judged of, to some extent, by this quality, taken in connection with the aspect of the free surface as left in casting. When these consist of copper, antimony, tin, etc., the metal is harder and brittler than when pure. Cast in a colored ingot-mould, the free surface or top is whiter than usual, with perhaps a yellowish hue, and more brilliant, while presenting the usual smooth and nicely rounded appearance. If mixed with lead, the free surface, which is not so flush, exhibits a dull, flaky opacity, with little lustre. When pure, the free surface is round and smooth, and quite dark with a purplish tint. The cut surface, or the sides laying in contact with the ingot-mould, are whiter, having the characteristic bright purplish gray color. This difference in hue is permanent, notwithstanding the brilliancy be lost by exposure to the atmosphere. Its fusibility also affords some criterion, being diminished by antimony, copper and most other metals, but increased by lead and tin, although the metals might be such as to counteract each other in this respect.

Bismuth is not at all affected by water in any condition, but it soon tarnishes on exposure to the air, although the tarnish is slight, amounting to little more than loss of lustre. Nitric acid is its proper solvent, and acts upon it with great vehemence, giving rise to dense orange colored fumes. Prof. Piggot says, that the contact of platinum arrests this action, which it is hardly necessary to remark, is not the case.* Sulphuric acid, heated to the boiling point, effects its solution, though with difficulty; but when cold, it has little action upon

*"It [bismuth] is soluble in nitric acid and aqua regia, but the process is immediately arrested by laying a piece of platinum on the metallic bismuth." *Dental Chemistry and Metallurgy*, p. 413.

It is singular that such errors should be accepted *without examination*, and transmitted from author to author, when the simplest test would suffice to correct them.

it, amounting merely to a slight tarnish. The action of muriatic acid is also very feeble, not sensibly affecting it when cold, and but little when heated.

Commercial bismuth may be purified by dissolving it in nitric acid, precipitating by pouring the clear solution into distilled water, digesting the resulting subnitrate with caustic potash to remove remaining traces of arsenic, and reducing with a flux prepared by fusing together one part of nitre and two parts of bitartrate of potash. Although the process of purification may be troublesome—(the theory is simple enough) —it can not be safely dispensed with, if the metal is designed as a constituent of alloy, for use in the mouth; for notwithstanding this metal, when pure, may be regarded innocuous as to any constitutional effects that could result from this use of it, it is more liable to contain, in its crude state as sold in commerce, pernicous contaminations than most others, which is more to be apprended at the present time when the enormous price of the article furnishes such temptation to adulteration.

As an ingredient in alloys bismuth has long been pre-eminent among metals for its property of promoting fusibility. The most remarkable instance of this is afforded in certain combinations with lead and tin, distinguished as "Fusible Metal," also called Newton's alloys, from the original discovery; also Rose's, or Darcet's metal, the former having made a supposed improvement in the formula, and the latter having reproduced it in France for dental purposes. It is adduced in all the chemical text books to illustrate the effect of combination in promoting fluidity and the property of bismuth as a fluidifying agent. "A remarkable instance," says Prof. Ure, "of fusibility increased by mutual affinity in their state of combination is afforded in the fusible metal, consisting of eight parts of bismuth, five lead and three tin, which melts at the heat of boiling water or 212° Fahr., though the melting point deduced from the mean of its components should be 514°. This alloy may be rendered still more fusible by adding a very little mercury to it, when it forms an excellent material for certain anatomical injections, and for filling the hollows of carious teeth." *Ure's Dict., etc.* Although different formulas are given for this alloy, (3 parts tin, 5 lead, 8 bismuth ;—2 tin, 3 lead, 5 bismuth ;—1 tin, 1 lead, 2 bismuth, etc.,) they are all substantially the same in properties, melting at a similar temperature, being fluid at about 200° Fahr., when the bulb of the thermometer is immersed in the melted metal, and if tested in water, varying from 205° to 208°. Heating in water does not, according to my experience, indicate as low a melting point, nor as uniform results, in the case of these, or of other fusible alloys, as when the other mode is adopted.* Since in the heating of water, the lower portion becomes hot first, and there is several degrees' difference of temperature in different strata, until the full boiling point is reached, it is important, if the test is conducted in this medium, that the metal be placed by the side of the thermometer bulb, *at the same depth in the fluid*, and not in a dish upon its surface, exposed to the cool atmosphere, much less in a porcelain cup, a watch-glass, or other non-conductor.†

The rule to be observed for attaining the greatest fusibility in the combination of these metals requires that the amount of lead be at least equal to that of tin, and not to exceed double the amount; and that the quantity of bismuth be equal to the sum of the other two.

This combination afforded the most fusible alloy known until quite recently. (Of course the addition of mercury, itself fluid at 39° below zero, lowers its melting point in proportion to the quantity added, by simply communicating its own fluidity to the mixturs, but without imparting any new property, forming not an alloy proper, but an *amalgam.*)

---

*For remarks on "Determining the Melting Point of Metals," see *Journal of the Franklin Institute*, Vol. 43, page 61; also copied in the *Dental Cosmos* for February 1862.

†See "An Experiment," in the last number *American Dental Review.*

But within the past four or five years, three distinct alloys have been added to the list, one being but slightly less fusible than that, and two much exceeding it in this property; the discovery of all of which happened to fall to the writer of this paper.

The first discovered (June, 1858) consists of the *three* metals, bismuth, tin and cadmium. The most fusible proportions of this alloy appears to be, three parts bismuth, one tin, and one cadmium, although a little increase of other of the two last named metals does not alter the result. It is fluid around the bulb of the thermometer at about 210° and congeals between 200° and 204°. Tested in hot water a higher melting point is indicated. The next, (discovered same date) consists of the *four* metals, bismuth, tin, lead and cadmium, forming the most fusible alloy we have, which is well enough known, having been repeatedly referred to in the scientific journals. It is fluid at 150°, and congeals at the same degree. Melted in water, it fuses between 150° and 160°, and is hard at 150°. Prof. Silliman gives its melting point at "about 158°," but Lipowitz puts it as low as 140°. Perhaps my own measurements express it as nearly as any, being about the mean between the two, and the result of carefully repeated tests. The last consists of the *three* metals bismuth, lead and cadmium. In October, 1858, I noted that two parts of bismuth, one of lead and one of cadmium, melt at a heat so low as to *soften* (without becoming fluid,) in boiling water; and again, (April, 1859,) that four parts bismuth, two lead and one cadmium, became *fluid* at the same heat; but though noting the fact for further inquiry, neglected to follow it up at the time, and it finally slipped my memory; until November, 1861, when on contrasting the two notes, I resolved to trace up the ultimate result, which proved the most fusible combination of these metals to consist of seven parts bismuth, six lead and one cadmium; forming an alloy fusible at 180°, (or, in water, a few degrees higher,) being the most fusible alloy known that consists of but *three* metals,—a most remarkable result, considering the small proportion of cadmium employed, and the high melting point indicated by the mean of the constituents.* Add to these the production by the means of cadmium, of another very fusible alloy *without bismuth*, (and therefore out of place to notice here, but heretofore described) and which, though less fusible than the preceding, melts at a lower temperature than any other not containing the latter metal, or containing only a proportion of its equivalent to that employed of the former; we find that bismuth, which has so long occupied the front rank as a fluidifying agent in alloys, must henceforth share the place with cadmium.

Bismuth not only confers fusibity but prevents shrinkage in alloys particularly of lead and tin: the fusible alloy referred to actually expands. This makes the metal a valuable adjunct in the formation of dies and counter-dies for use in mechanical dentistry. Its alloy with tin is harder than either of the constituents. Its alloys with lead are in hardness about like bismuth when the latter is in excess, but when lead predominates, softer, and in all cases harder than lead. In malleability and flexibility they are nearly the mean between the two metals, possessing these qualities always in a greater degree than bismuth, but less than lead. Its alloys with lead are much more subject to oxidation than either of the components, while its alloys with tin alone, or with lead and tin, are not.

It does not alloy with zinc, the metals separating in distinct portions, each, however, retaining a very small percentage of the other. But if tin be present in any considerable proportion, it serves as a medium of combination, and we have a homogeneous alloy. With antimony it alloys perfectly, the result being hard and brittle.

Dr. Piggott, in his work on *Dental Chemistry and Metal-*

---

*A description of this alloy was published in the *Journal of the Franklin Institute*, January, 1862. And also in Silliman's *American Journal of Science and Art*, for March, which see.

*lurgy*, makes some singularly contradictory statements in regard to bismuth. In one place he says: "Bismuth, zinc and antimony, alloyed with lead, *cause it to contract* on cooling;"—(whereas bismuth and antimony serve to prevent the contraction of lead, while lead will not alloy with zinc, whether alone or combined with these metals.) In another place he says more correctly: "Bismuth increases the fusibility of an alloy, and, if added in sufficient quantity, usually *confers* on the compound the property of *expansion*. The dentist (he adds) uses it to *alloy his zinc* for moulds, on account of the two properties above mentioned." But in another place he states that *zinc does not alloy* "at all with bismuth."—But judging from the errors throughout the work in respect to the properties of metals, the auther had little practical knowledge of this part of his subject, taking the statements of others, however, discrepant, on trust, without stopping to examine—a failing, by the way, which would appear to be the rule, and not the exception, among most authors when treating of the properties and relations of metals in respect to each other.

With gold, silver, copper and other of the less fusible metals, bismuth forms brittle compounds that are worthless for use. Its amalgams are fluid at a low temperature. It conducts heat less readily than most metals. It occurs native, and sometimes as an oxide; also as a sulphuret, and is found associated with sulphurets of cobalt, arsenic, copper, lead nickel, tellurium, etc. It is obtained principally from Saxony.

Bismuth has been employed to a considerable extent in operative dentistry. It constituted a large proportion, (one half,) in the fusible alloy, which, under the name of Darcet's metal was so generally resorted to several years ago for filling teeth, but which was finally abandoned, owing partly to the difficulty of introducing it perfectly and partly to the alloy's requiring the addition of mercury to render it fusible at a heat sufficiently low for the purpose, which rendered it vulnerable to the same objections that were urged against the amalgam pastes, while the latter could be much more easily and effectually manipulated. It forms an important constituent in all the alloys used for making artificial dentures upon the cheoplastic plan. It enters largely as an ingredient in the plastic metallic filling, lately introduced as a substitute for the ordinary base metals heretofore resorted to.

Its employment in the laboratory in the formation of dies and moulds, always somewhat limited on account of the cost, is likely to be precluded altogether by the présent greatly advanced rates.

Until recently the wholesale price of commercial bismuth in Europe was, according to the New American Cyclopædia, from 30 to 40 cents per pound; and imported into England 70 dollars per cwt. It retailed in this country at about one dollar and a quarter a pound; but for the past few years sold at a dollar and a half. About the first of the present year its price rose to three dollars per pound. By the latter part of July it had risen to five dollars, and gradually advanced to ten dollars. The last accounts it had advanced still higher, and fears were entertained that ere long it would be difficult to procure at any price, the whole annual product of the mines not exceeding ten or twelve thousand pounds. It may, therefore, now be ranked among the "precious metalls" — a place to which, considering that it resists the strong acids as well as silver or better, it really does not discredit. It is to be hoped that its great value will stimulate its procurement from other localities where it is found, but where its production heretofore has not proved remunerative.

New York, Dec., 1862.

[TO BE CONTINUED.]

